# Epigenetic Biomarkers of Cardiovascular Risk in Frail Patients—A Scope Review

**DOI:** 10.3390/cimb47060422

**Published:** 2025-06-05

**Authors:** Stanisław Wawrzyniak, Julia Cieśla, Magdalena Woś, Ewa Wołoszyn-Horák, Michał M. Masternak, Tomasz Kukulski, Ewa Stępień, Andrzej Tomasik

**Affiliations:** 1 Doctoral School, Medical University of Silesia, Poniatowskiego 15, 40-055 Katowice, Poland; d201250@365.sum.edu.pl; 2 Department of Internal Medicine and Clinical Pharmacology, Medical University of Silesia, Medyków 18, 40-752 Katowice, Poland; magdalenawos94@gmail.com; 3 II Department of Cardiology, Faculty of Medical Sciences in Zabrze, Medical University of Silesia, Marii Skłodowskiej-Curie 10, 41-800 Zabrze, Poland; krystyna.woloszyn@interia.pl (E.W.-H.); tkukulski@sum.edu.pl (T.K.); 4 Department of Biomedical Sciences, Burnett School of Biomedical Sciences, College of Medicine, University of Central Florida, 6900 Lake Nona Blvd, Orlando, FL 32827, USA; michal.masternak@ucf.edu; 5 Department of Medical Physics, Marian Smoluchowski Institute of Physics, Jagiellonian University, 30-348 Kraków, Poland; e.stepien@uj.edu.pl

**Keywords:** frailty, epigenetic biomarkers, microRNA, methylation, epigenetic clock, cardiovascular disease, cardiovascular risk

## Abstract

Epigenetic biomarkers offer promising potential for early identification and risk stratification of frail individuals susceptible to adverse cardiovascular outcomes. This scope review aimed to identify and evaluate epigenetic biomarkers concurrently associated with frailty and increased cardiovascular risk, potentially facilitating more precise patient stratification and treatment decisions. A two-stage literature search was performed using PubMed, Scopus, Web of Science, and Embase databases from the year 2000 through 27 December 2024. Stage 1 identified studies reporting epigenetic biomarkers associated with frailty in blood-derived human samples. Stage 2 assessed cardiovascular relevance by screening the frailty biomarkers identified in Stage 1 for their documented association with cardiovascular diseases. Two independent reviewers conducted screening, data extraction, and risk-of-bias assessments, resolving disagreements via a third reviewer. The primary outcomes were the association of biomarkers with frailty severity and cardiovascular risk. Key epigenetic biomarkers identified included microRNAs (particularly miR-21, miR-146a, miR-451, and miR-92a) and DNA methylation markers (LINE-1 methylation, epigenetic clocks like GrimAge and DunedinPACE, and possibly novel, emerging clocks like DNAmCVDscore and the Smoking Index). Due to specificity limitations, these biomarkers are most promising when used collectively as part of multimarker panels rather than individually. Future research should validate multimarker panels, explore novel biomarkers, and assess clinical integration to optimize precision medicine in frail cardiovascular populations.

## 1. Introduction

Frailty is a multidimensional clinical syndrome characterized by diminished strength, endurance, and physiological function, which increases vulnerability to stressors and adverse health outcomes. It has emerged as a significant predictor of mortality and disability in either dwelling population [1,2], or particularly, in older adults and those with chronic diseases such as cardiovascular disease (CVD) [3,4,5,6,7].

When we review Linda Fried’s seminal paper on frailty [1,2,7,8,9,10,11,12,13,14,15,16,17,18,19,20,21,22,23], we observe that the overall mortality rate increases with the progression of frailty from robust to frail, along with the increasing incidence of cardiovascular disease (CVD) in these subgroups. The prevalence of angina at baseline was 14.5% in non-frail, 21.0% in intermediate, and 28.8% in the frail population [2]. Respectively, the history of previous myocardial infarction was reported by 7.3, 10.3, and 13.3% of the populations mentioned above. The prevalence of congestive heart failure was 2.0, 4.5, and 13.6% of the population, as discussed above. The mortality rate in this community-dwelling population was 3.0% for non-frail patients at 3 years of follow-up and reached 43% for frail patients at 7 years of follow-up [2]. To complete the phenotype picture of frail patients, they have a more significant comorbid burden and angiographic disease severity [8], they are known to more frequently present the highly vulnerable thin cap fibroatheroma within coronaries [9], and they have increased bleeding risk [10]. None of the frailty assessment tools has been proven superior to others, and the authors of the above-cited papers have utilized the Fried frailty scale, clinical frailty scale, hospital frailty risk score, or frailty index (FI). Each of the frailty tools is capable of identifying the frail subpopulation at risk of adverse outcomes for institutionalization, overall mortality, and procedural adverse outcomes [11]. A novel approach introduced by the REPOSI study employed cluster analysis to define four patient clusters based on comorbidity, independence, and cognition, with certain clusters exhibiting notably higher short-term mortality [12,19]. Ratcovich et al. [13] have reported no difference between the Clinical Frailty Scale and the Global Registry for Acute Coronary Events (GRACE) in predicting the 12-month mortality of frail patients. Apart from frailty as a risk factor, the patients vary in age, sex, comorbidities, and clinical presentation, making the treatment assignment challenging.

According to Afilalo et al. [14], we lack optimized resource allocation, which would allow frail patients to avoid costly but futile interventions. Eventually, frailty should not be viewed as a reason to withhold care but rather as a means to deliver it in a more patient-oriented way [15]. This raises critical questions: How can we better identify frail patients who may benefit from interventions? Who are the likely responders, and how should care be personalized? Identifying biomarkers would allow cardiologists to predict the functional trajectories of older adults at preclinical stages [16]. Precision medicine was defined by the National Research Council’s Toward Precision Medicine in 2008 as: “The tailoring of medical treatment to the individual characteristics of each patient … to classify individuals into subpopulations that differ in their susceptibility to a particular disease or their response to a specific treatment. Preventive and therapeutic interventions can then be concentrated on those patients who will benefit, sparing expense and side effects for those who will not”.

Epigenetics is a rising discipline in biomedicine that aims to improve predictive and precision medicine by discovering new mechanisms underlying diseases and providing new biomarkers [17,18]. It refers to heritable changes in gene expression that do not involve changes to the DNA sequence itself, such as DNA methylation, histone modifications, and non-coding RNA regulation. These mechanisms play an essential role in cellular aging and disease pathogenesis, including frailty and CVD. Despite increasing recognition of their significance, the interplay between epigenetic modifications and frailty remains underexplored.

Non-coding RNAs, including microRNAs (miRNAs) and long non-coding RNAs (lncRNAs), are key regulators of gene expression that do not encode proteins but play essential roles in various physiological and pathological processes.

MicroRNAs (miRNAs) are small, ubiquitous, and non-coding RNA molecules. By base-pairing with complementary sequences within mRNA molecules, miRNAs post-transcriptionally inhibit protein translation by silencing or allowing degradation of mRNA. Thus, miRNAs act as powerful regulators of gene expression at the post-transcriptional level [19]. The importance of this regulation route is significant since about 60% of the human genome is miRNA-regulated. More than 1000 miRNAs are expressed in human cells. Their functions spread between housekeeping molecules tissue-specific or cell-type-specific regulators, and it has become increasingly clear that only a relatively small number of them have key functions [20]. MiRNAs molecules are detectable in varied body fluids, including blood, since they are protected from RNase activity by microvesicles such as exosomes forming protein complexes with Ago2 and lipoproteins. There are many papers providing evidence on the regulatory role of miRNAs in inflammation processes [21,22,23], including autoimmunity [24] and CVDs [25,26]. Since they are involved in different cellular processes, their deregulation leads to different diseases and physiological processes, including aging, sarcopenia, age-related musculoskeletal impairments, cancer, and neurodegenerative diseases. In the development and progression of CVD, they play multifaceted roles. Dysregulated miRNAs contribute to endothelial dysfunction, vascular inflammation, oxidative stress, and fibrosis—key drivers of atherosclerosis and other CVD pathologies. MiRNAs also participate in cardiac remodeling processes, influencing cardiomyocyte hypertrophy, fibroblast activation, and extracellular matrix deposition [26]. The regulatory role of some miRNAs, variability in expression of certain miRNAs throughout life, and the simplicity of their blood level measurement suggest great potential of miRNAs as biomarkers.

Long non-coding RNAs (lncRNAs) are a diverse group of non-protein-coding transcripts longer than 200 nucleotides. They regulate gene expression through multiple mechanisms, including chromatin remodeling, transcriptional control, and post-transcriptional modulation. LncRNAs have emerged as potential biomarkers of frailty due to their regulatory role in aging, muscle function, and cellular senescence [27]. They are involved in numerous biological functions such as cell differentiation, inflammation, and stress response, suggesting their potential role as biomarkers and therapeutic targets.

DNA methylation (DNAm) refers to the addition or removal of methyl groups to Cytosine-phospho-Guanine (CpG) sites. Long Interspersed Nuclear Elements-1 (LINE-1) are repetitive sequences that make up approximately 17% of the human genome. Due to their high CpG content and widespread distribution, LINE-1 elements are often used as surrogate markers of global DNA methylation. Although LINE-1 methylation does not capture all genomic regions, it provides a cost-effective and widely accepted approximation of overall methylation levels, particularly in epidemiological and aging research [28].

Epigenome-Wide Association Studies (EWAS) have emerged as a crucial tool in identifying DNA methylation patterns associated with aging and frailty. These studies analyze genome-wide methylation changes to uncover epigenetic signatures linked to functional decline and age-related health outcomes. The findings from multiple EWAS studies have provided evidence supporting the role of specific CpG sites as potential biomarkers of frailty.

A large body of literature provides evidence of chronological age being the main factor of genome-wide methylation [29,30,31,32], but the biological processes vary between individuals, and the chronological age is not entirely overlapping with biological age in many cases. Epigenetic Age Acceleration (eAA) refers to the discrepancy between predicted epigenetic age (calculated from an epigenetic clock) and chronological age. A positive eAA means that an individual’s epigenetic age is higher than their chronological age, which is often interpreted as faster biological aging. The reasons for such differences are still elusive, but there are many known factors that affect the eAA, such as inherited genetic factors as well as body mass index, diabetes, sex, cardiovascular diseases, smoking, and many more [33]. Hence the morbidity strongly correlates with the eAA, and a DNAm level advance is a well-known all-cause-mortality predictor [34,35].

Epigenetic clocks are machine learning-based tools that estimate DNAm levels at specific CpG sites, unique to each clock. First-generation epigenetic clocks, such as the Hannum clock and Horvath clock, are highly effective in predicting chronological age with near-perfect accuracy [36,37]. However, their utility as mortality assessment tools is limited due to their lack of integration with environmental and health-related factors [29,38,39,40]. Second-generation clocks, PhenoAge [41] and GrimAge [42], designed to address these limitations, incorporate clinical data to enhance biological age estimation. PhenoAge evaluates DNAm in conjunction with ten clinical markers—chronological age, albumin, creatinine, glucose, C-reactive protein (CRP), lymphocyte percentage, mean cell volume, red blood cell distribution width, alkaline phosphatase, and white blood cell count—using a penalized regression model [29]. GrimAge, on the other hand, integrates DNAm-based surrogates for physiological and stress-related biomarkers, including smoking pack-years and plasma proteins such as adrenomedullin, CRP, plasminogen activation inhibitor 1 (PAI-1), and growth differentiation factor 15 (GDF15). Different clocks produce different measures of eAA, so HorvathAA, HannumAA, GrimAgeAA, and PhenoAgeAA refer to epigenetic age acceleration calculated using those respective clocks.

In this review we evaluate evidence for epigenetic biomarkers that concurrently indicate frailty and cardiovascular risk, with the goal of enhancing stratification and guiding targeted interventions in this vulnerable population [17,18].

## 2. Materials and Methods

The study follows the PRISMA 2020 guidelines to ensure a systematic and transparent approach in literature selection and data synthesis (the PRISMA 2020 checklist can be found in the Supplementary Materials). The analysis was conducted in two stages.

### 2.1. Stage 1: Identification of Epigenetic Biomarkers of Frailty

Studies were included if they investigated epigenetic biomarkers (DNA methylation, histone modifications, or microRNA expression) associated with frailty syndrome in human populations. Only studies using blood-derived samples (whole blood, plasma, or serum) were considered. Studies published in English between 2000 and 2024 were included.

### 2.2. Stage 2: Screening for Cardiovascular Relevance

The criterion for selecting biomarkers with clinical relevance was based on a two-step evaluation process conducted after their identification in the initial stage of the study. First, we assessed the quantity and quality of existing literature investigating the association between each biomarker and cardiovascular disease (CVD), with a particular emphasis on large population-based studies and those with a low risk of bias. Notably, many biomarkers were excluded at this stage due to the limited number of high-quality studies supporting their relevance. Second, we carefully reviewed the reported findings and conclusions regarding the biomarker’s relationship with CVD outcomes. Only those biomarkers supported by consistent and robust evidence were considered clinically relevant for further analysis. This step ensured that only biomarkers with dual relevance to frailty and CVD were considered.

### 2.3. Exclusion Criteria

Studies focusing only on non-blood tissues (e.g., cardiac tissue, vascular endothelium). Animal or in vitro studies. Reviews, meta-analyses, and case reports without original data. Studies without a clear assessment of frailty or CVD risk.

### 2.4. Information Sources

A comprehensive literature search was conducted using the following databases: PubMed, Scopus, Web of Science, and Embase. Additionally, reference lists of relevant systematic reviews and original studies were manually screened. The final search was conducted on 27 December 2024.

### 2.5. Search Strategy

For each stage, different search strategies were applied:

### 2.6. Stage 1 (Epigenetic Biomarkers of Frailty)—The Search Methodology for Each Database Is Presented in Figure 1

### 2.7. Stage 2 (CVD Relevance Search for Selected Biomarkers)

(“selected biomarker”) AND (“cardiovascular” OR “cardiovascular disease*” OR “coronary artery disease” OR “myocardial infarction” OR “heart failure”).

Filters: English language, human studies.

### 2.8. Selection Process

Stage 1: Two independent reviewers screened titles and abstracts to identify epigenetic biomarkers of frailty.

Stage 2: The most relevant frailty biomarkers were screened for CVD association in a second literature search.

Disagreements were resolved by a third reviewer.

### 2.9. Data Collection Process

Two reviewers independently extracted data using a standardized form (Table 1 and Table 2 presented in the Results section). Extracted data included study characteristics, biomarker type, and population details.

### 2.10. Primary Outcomes

The number of studies directly linking cardiovascular disease biomarkers with the frail population I extremely limited, highlighting a gap in the current research landscape.

(1) Association of biomarkers with frailty severity.
(2) Association of selected biomarkers with CVD risk.

For each primary outcome defined, all available results compatible with the specified outcome domains were sought from included studies, regardless of the measures used, time points, or statistical analyses. When multiple results concerning the same biomarker were available within a single publication, we selected those considered clinically most relevant (e.g., longest follow-up duration, largest study population, or best-validated outcome measure).

### 2.11. Other Variables

Each study was also assessed for demographic data (age, sex, comorbidities), laboratory methods used for biomarker detection, and frailty assessment tools. In cases of missing or unclear information regarding the listed variables, data were categorized as ‘not reported’. No assumptions or imputations were performed for incomplete data unless explicitly stated within the original publications.

### 2.12. Risk of Bias Assessment

The risk of bias assessment was conducted using a qualitative, structured approach that considered key methodological domains for each study. Factors included sample size, study design (cross-sectional vs. longitudinal), quality and objectivity of outcome measures (e.g., self-reported vs. biologically measured data), control for potential confounders, and the representativeness of the study population. Studies were assigned a low, moderate, or high risk of bias based on the cumulative presence of limitations across these domains. While the classification was qualitative, it followed consistent criteria applied uniformly across all included studies.

### 2.13. Effect Measures

Effect measures (odds ratios, hazard ratios, mean differences, AUC) were not directly calculated or quantitatively synthesized in this scoping review. Instead, these metrics were qualitatively interpreted based on results reported in the included studies.

### 2.14. Methods of Data Synthesis and Rationale

Eligibility of studies for inclusion into each synthesis stage was determined based on predefined criteria (biomarker type, relevance to frailty, cardiovascular relevance, type of biological sample). Study characteristics were systematically tabulated (Table 1 and Table 2) and compared against these criteria independently by two reviewers, and consensus was achieved by resolving disagreements with a third reviewer. No data conversions or imputations were performed, and no special methods were required to handle missing summary statistics, as results were presented and synthesized qualitatively based on available published data.

**Figure 1:**
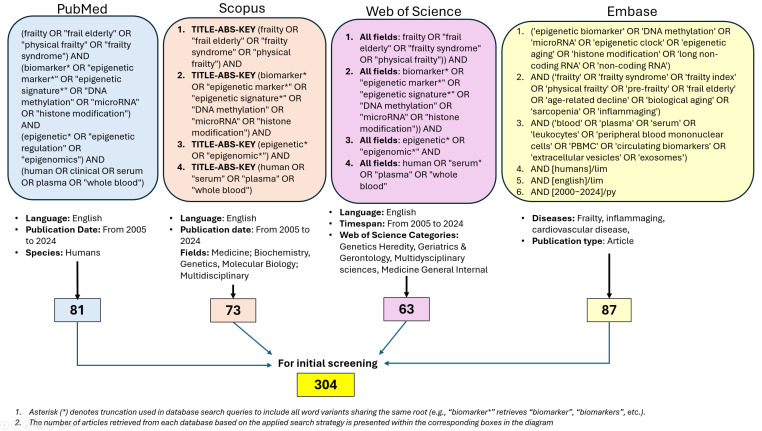
Data bases screening.

**Figure 2:**
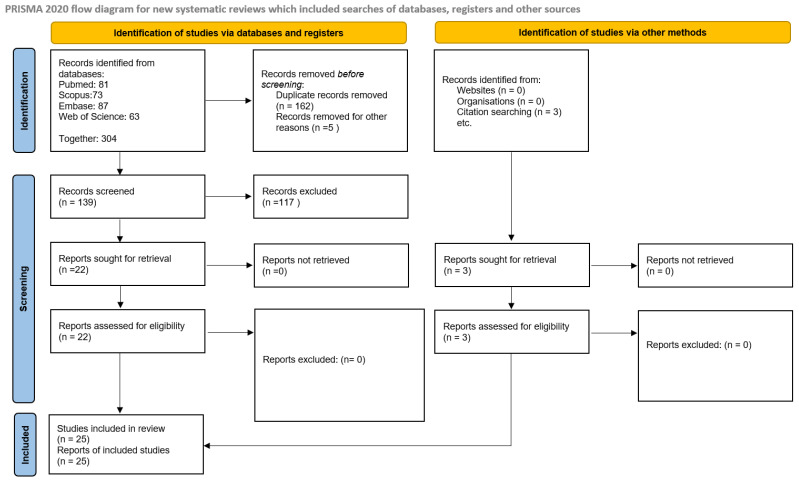
Summary of selected studies according to PRISMA 2020 flow diagram.

Extracted data were summarized in structured tables (Table 1 and Table 2) presenting individual study characteristics, biomarker details, and associations identified. Visual summaries of the literature search and selection process were presented in Figure 1 and Figure 2 (PRISMA 2020 flow diagram). No additional graphical methods for synthesizing or displaying results were applied.

Due to the substantial methodological heterogeneity, variability in biomarkers assessed, differences in outcome measures, and limited number of directly comparable studies, a meta-analysis was not performed. Instead, a qualitative narrative synthesis of the results was undertaken. The narrative synthesis involved summarizing and interpreting findings according to biomarker type, biological plausibility, and reported associations with frailty and cardiovascular outcomes. This approach was selected to comprehensively integrate the available evidence, given the heterogeneity and complexity of the included studies. Given that quantitative synthesis (meta-analysis) was not conducted, statistical methods to explore causes of heterogeneity among studies (e.g., subgroup analyses, meta-regression) and sensitivity analyses to assess robustness of the synthesized results were not performed. The formal methods to assess the risk of bias arising from missing results (reporting biases), such as funnel plots or statistical tests, were not applicable. Instead, the potential impact of missing results was qualitatively considered in the interpretation and discussion of findings.

### 2.15. Certainty Assessment

Formal methods to assess the certainty or confidence in the body of evidence for each outcome were not applied due to the narrative and exploratory nature of this review. Instead, the limitations of the available evidence and potential implications for certainty of conclusions were qualitatively considered and explicitly discussed.

## 3. Results and Discussion

### 3.1. Identification of Epigenetic Biomarkers of Frailty

Identifying relevant epigenetic biomarkers within the broad and heterogeneous group of cardiovascular disease biomarkers proved to be significantly more challenging than focusing on the narrower set of frailty-related biomarkers. At the same time, there is a striking lack of studies directly comparing biomarkers in the context of both frailty and cardiovascular disease, highlighting the novelty and necessity of this research. Therefore, our methodology was designed to first identify epigenetic biomarkers of frailty and subsequently assess their relevance to CVD.

Screened frailty biomarkers are presented in Table 1.

**Table 1:** Epigenetic Biomarkers of Frailty.

Epigenetic Factor	Reference Article	Tested Biomarker	Result	Characteristics and Bias Risk
**MicroRNA**	[22]	miR-21	Mir-21 was increased in frailty elders.	Cross-sectional study; *n* = 96 (22 aged without frailty, 34 aged with frailty, 40 young controls). Moderate risk of bias due to limited cohort size, potential confounding factors, and lack of longitudinal validation.
		miR-146a	MiR-146a was increased in robust elders.	
		miR-223 miR-483	Mir-233 and mir-483 were increased in both groups.	
**MicroRNA**	[23]	miR-21 (selected from 365 miR tested)	Increases with age and in cardiovascular disease.	Cross-sectional study; *n* = 150 (Validation cohort: 111 healthy controls, including 30 centenarians; 15 healthy centenarian offspring; 34 CVD patients); Moderate risk of bias due to limited cohort size, heterogeneity of age groups, potential confounding factors, and lack of longitudinal validation.
**MicroRNA**	[43]	miR-10a-3p, miR-92a-3p, miR-185-p, miR-194-5p, miR-326, miR-532–5p, miR-576–5p, miR-760	Increased in frail elders.	Cross-sectional study. High risk of bias due to very limited sample size, (*n* = 14; 7 robust vs. 7 frail). Potential confounders not accounted for, lack of longitudinal validation.
**MicroRNA**	[44]	miR-151a-5p, miR-181a-5p miR-1248	Decreased in older subjects.	Cross-sectional study. High risk of bias due to small sample size (*n* = 22; 11 young vs. 11 old), limited age range (30–64 years old), lack of longitudinal validation, and potential confounders not addressed.
		miR-21	No differences between old and young.	
**MicroRNA**	[45]	miR-451a	Increased in frail subjects.	Cross-sectional study with intervention component—evaluation of miRNA expression before and after 12-week multicomponent exercise protocol (VIVIFRAIL) in frail (*n* = 50) and robust (*n* = 136) subjects. Subgroup analysis of participants undergoing physical exercise intervention: 15 frail and 30 robust. Moderate risk of bias due to non-randomized assignment, differences in population size between groups, and potential confounding from other health conditions not accounted for. Lack of longitudinal follow-up beyond exercise protocol.
**lncRNAs**	[46]	9p21-23 locus (ANRIL)	ANRIL expression is reduced in frail individuals, which may lead to dysregulation of CDKN2A/B, key genes involved in cellular senescence and inflammation.	Large cohort study (*n* = 637) of Ashkenazi Jewish adults aged 65+. Moderate risk of bias due to selection bias (limited to Ashkenazi Jewish population), lack of randomization, and potential measurement bias related to subjective grip strength assessment.
**Methylation (EWAS)**	[47]	CpG sites: cg19283806 (KCNQ1), cg21572722 (PHF21A), cg05575921 (AHRR),	Hypomethylation at these CpG sites was associated with increased frailty scores.	Observational study (*n* = 346 twin participants, aged 65–91 years; 128 analyzed for DNA methylation). Moderate risk of bias due to selection (participants from a specific twin registry), potential measurement bias (self-reported medical history), and no adjustment for all confounders. However, familial relatedness was accounted for.
**Methylation (EWAS)**	[48]	CpG site located in the MAF1 gene	Positive correlation with physical frailty phenotype.	Cross-sectional study (*n* = 791, All participants aged 70). Moderate risk of bias due to cross-sectional design, potential confounding factors, and limitations in the sample (incomplete data on frailty).
**Methylation (EWAS)**	[49]	eFRS	Predicts frailty prevalence and incidence over time.	Large observational study with cross-sectional and longitudinal validation; *n* = 3986 (ESTHER and KORA-Age cohorts). Moderate risk of bias due to the self-reported frailty index components, potential confounding factors (e.g., smoking, BMI, education), and voluntary recruitment.
**Methylation (EWAS)**	[50]	589 CpG sites and 3 differentially methylated regions (DMRs)	Differentially methylated in association with frailty and biological aging; supporting the role of epigenetic clocks in risk prediction.	Meta-analysis of four twin cohort studies: SATSA 450K (*n* = 379) and EPIC (*n* = 146); LSADT 1997 (*n* = 304) and 2007 (*n* = 86). Moderate risk of bias due to potential attrition and limited generalizability to non-twin populations, possible differences in frailty measures across cohorts (self-reported data, slightly different items included in the Frailty Index in SATSA and LSADT), and the small sample size for certain subgroups (e.g., limited sample sizes in the LSADT 2007 cohort and SATSA EPIC, which may affect sex-specific or age-specific analyses). Longitudinal data help strengthen the robustness of the findings, but differences in cohort characteristics may introduce heterogeneity.
**Methylation** **(CpGlobal)**	[51]	Global methylation levels	Lower global DNA methylation levels were associated with increased frailty in middle-aged and elderly subjects; a decline in methylation over a 7-year period correlated with worsening frailty status.	Observational study with cross-sectional and longitudinal analysis; *n* = 318 (65–105 years old, Calabria, Italy). Moderate risk of bias due to lack of representativeness (single geographical region), differences in frailty assessment methods (HCA-based classification), and small sample size in the >90 age group.
**Methylation** **(LINE-1)**	[52]	LINE-1 DNA methylation	Lower methylation levels in males with sarcopenia in comparison to women.	Cross-sectional study; *n* = 204 (aged 60+ years, from a rural community in Japan). Moderate risk of bias due to moderately small sample size, potential confounding factors (e.g., smoking, BMI), and the specific rural population, which may limit generalizability to other regions.
**Methylation** **(LINE-1 and specific marker loci)**	[53]	LINE-1 DNA methylation	LINE-1 methylation showed no significant association with frailty.	Cohort study; *n* = 552 (Newcastle 85+ Study, participants aged 85). Moderate risk of bias due to the exclusion of individuals with cognitive impairment and the lack of complete methylation data for all participants; reliance on self-reported health data and the focus on a specific cohort (elderly individuals from the North East of England) may limit generalizability.
		Promoter-specific CpG island methylation: *EPHA10*, *HAND2*, *HOXD4*, *TUSC3* and *TWIST2*	Lower promoter-specific CpG island methylation levels were associated with reduced frailty.	
**Methylation** **(Epigenetic clock)**	[39]	GrimAge and PhenoAge	Positive correlation with frailty phenotype.	Cohort study; *n* = 490 (Irish Longitudinal Study on Ageing, TILDA cohort, participants aged 50+, follow-up up to 10 years). Moderate risk of bias due to the selective sample and the reliance on self-reported health data.
		Horvath and Hannum	No correlation with frailty.	
**Methylation** **(Epigenetic clock)**	[40]	Horvath	DNAmAge measures (Horvath’s DNAmAge, AgeDiff, AgeResid) did not predict mortality when adjusting for chronological age. Frailty index remains a more reliable indicator of biological aging.	Cohort study; *n* = 262 (Louisiana Healthy Aging Study, participants aged 60–103). Moderate risk of bias due to cross-sectional nature of the study and relatively small sample size restricted to only Caucasian participants, which may limit generalizability to other ethnic groups.
**Methylation** **(Epigenetic clock)**	[54]	Horvath Hannum Lin epiTOC PhenoAge DunedinPoAm GrimAge Zhang	GrimAge and PhenoAge showed positive correlation with frailty phenotype.	Large cohort study; *n* = 3222 (Rotterdam Study and Leiden Longevity Study, participants aged 30–98). Moderate risk of bias mostly due to the inclusion of only healthy individuals who could attend the research centers, potentially leading to selection bias. Additionally, reliance on proxies for missing frailty measures could impact the precision of the frailty scores.
**Methylation** **(Epigenetic clock)**	[55]	Horvath Hannum Lin Zhang Yang Dunedin PoAm PhenoAge GrimAge	GrimAge and Hannum showed the strongest correlation with both baseline frailty and with its time alterations.	Large cohort study; *n* = 1446 (Canadian Longitudinal Study on Aging, participants aged 45–86). Moderate risk of bias due to the study’s cohort being predominantly community-dwelling, the exclusion of participants with missing data at follow-up, which may lead to attrition bias, and the reliance on self-reported lifestyle factors such as diet, physical activity, and smoking status.
**Methylation** **(Epigenetic clock)**	[56]	GrimAge PhenoAge MRscore-8CpGs	All DNAm markers correlated with each other and FI and independently predicted all-cause and cause-specific mortality. MRscore-8CpGs showed the strongest predictive power.	Large cohort study; *n* = 1771 (ESTHER Study, participants aged 50–75). Moderate risk of bias due to the use of a cross-sectional, single cohort design; the MRscore algorithm was based on a microarray with missing CpGs, which may have impacted the predictive accuracy for mortality.
**Methylation** **(Epigenetic clock)**	[57]	GrimAge PhenoAge	Mediate the relationship between circulating metabolites and frailty.	Large cohort study; *n* = 980 (China Kadoorie Biobank, participants aged 50+). Moderate risk of bias due to rather young population, and the cross-sectional nature of baseline measurements, limiting causal inference between metabolites and DNAm aging.
**Methylation** **(Epigenetic clock)**	[58]	DunedinPACE	Higher DunedinPACE predicts subsequent increases in frailty.	Large cohort study; *n* = 524 (Swedish Adoption/Twin Study of Aging, participants aged 50–90). Moderate risk of bias due to potential attrition and limited generalizability to non-twin populations.
		Horvath Hannum PhenoAge GrimAge	Horvath, Hannum, PhenoAge and GrimAge showed no dynamic longitudinal association with frailty.	
**Methylation** **(Epigenetic clock)**	[59]	Epigenetic Age Acceleration (eAA) GrimAge PhenoAge Hannum Horvath	No significant relationship between eAA and changes in frailty over time; inconsistent associations across frailty models.	Cohort study; *n* = 395 (MOBILIZE Boston, participants aged 77–78). Moderate risk of bias due to short follow-up period (12–18 months) and limited sample diversity (all participants identified as white). Potential for regression to the mean in frailty measures.
**Methylation (Epigenetic clock)**	[60]	Horvath Hannum Skin & Blood PhenoAge GrimAge	No significant association was found between FI and DNAm age estimators.	Small cohort study; *n* = 31 (Italian semi-supercentenarians, aged 104–109). High risk of bias due to the small sample size and the homogeneity of participants, as all subjects were from a single region (Italy).
**Methylation** **and Telomere**	[61]	7-CpG Clock Horvath Hannum PhenoAge, GrimAge, Telomere Length	No significant longitudinal association between both telomere length and epigenetic clocks with functional decline. Cross-sectional analysis showed a weak association between only GrimAge and frailty.	Cohort study with cross-sectional and longitudinal components; *n* = 1083 (Berlin Aging Study II, participants aged 60–85). Moderate risk of bias due to potential sample selection (convenience sample), attrition over time, and limited generalizability to populations with higher morbidity.
**Methylation** **and Telomere**	[62]	Horvath Telomere Length	Frailty is significantly associated with the epigenetic clock (Horvath DNAm age acceleration), but not with telomere length.	Cohort study with cross-sectional components; *n* = 1820 (ESTHER cohort, participants aged 50–75). Moderate risk of bias due to the cross-sectional nature of the analysis, potential selection bias from the case-cohort design, and limitations from self-reported data.
**Methylation** **and Smoking**	[63]	Smoking Index (SI)—Smoking-related DNA methylation (CpG sites: MYO1G, GPR15, GNG12, CPOX, POLK, ALPP)	Smoking-related CpG sites were significantly associated with frailty. DNA methylation-based smoking indices correlated with frailty more strongly than self-reported smoking.	Cohort study with cross-sectional components; *n* = 1509 (ESTHER cohort, participants aged 50–75). Moderate risk of bias due to the cross-sectional design and potential confounding factors related to self-reported smoking status and health-related behaviors. The study benefits from validation in an independent sample and detailed adjustment for various covariates such as alcohol consumption and leukocyte distribution.

lncRNA—long non-coding RNA; miR—microRNA; DNAm—DNA methylation; LINE-1—Long Interspersed Nuclear Element-1; EWAS—Epigenome-Wide Association Study; CpG—cytosine-phosphate-guanine site; eFRS—Epigenetic Frailty Risk Score, based on methylation levels at 20 CpG sites; ANRIL—antisense noncoding RNA in the INK4 locus.

#### 3.1.1. MicroRNA

Among microRNAs, miR-21 is one of the most widely studied inflammation-related miRNAs and has been consistently implicated in aging and frailty. Mechanistically, circulating miR-21 plays a crucial role in pro-inflammatory signaling, as it can bind to toll-like receptors (TLRs) on immune cells, triggering NF-κB pathway activation and subsequent secretion of pro-inflammatory cytokines such as IL-6 and TNF-α [22]. Additionally, miR-21 directly modulates TGF-β signaling, a key pathway interconnecting inflammation, senescence, and cancer [64]. It is significantly elevated in frail individuals compared to both senile and young controls in the Rusanova et al. cohorts [22]. These findings align with results published by Olivieri et al. [23], where the authors applied exploratory factor analysis (EFA) to analyze 365 circulating miRNAs in young (20–65 years old) and old (66–95 years old) cohorts. Their analysis identified miR-21 as one of the most promising biomarkers of aging due to its progressive increase with age and additionally observed its higher expression in patients with CVD and systemic inflammation. Notably, while Rusanova et al. [22] is the only study that directly links miR-21 with frailty, the assessment of Olivieri et al. [23] and Hooten et al. [44] cohorts suggests that increased miR-21 expression is primarily observed among very old adults (the cohort of the Hooten et al. study had a mean age of approximately 64 years, making it significantly younger than the cohort studied by Olivieri et al.) and in those affected by inflammatory conditions, including CVD [23]. Given that frailty is widely recognized as a state of chronic low-grade inflammation and an age-related inflammatory syndrome (inflammaging), this strengthens the case for miR-21 as a potential biomarker of frailty and its progression.

MiR-146a is a component of a negative feedback loop that downregulates the levels of interleukin-1 receptor-associated kinase 1 (IRAK1) by repressing TNF receptor-associated factor 6 (TRAF6) and IRAK1 expression [65]. An increase in miR-146a concentration in cells with high senescence-associated secretory phenotype (SASP) leads to upregulation of inflammatory factor production [66]. Cellular senescence occurs as a response to excessive extracellular or intracellular stress. The senescence program locks the cells into a cell-cycle arrest that prevents the spread of damage to the next cell generation and precludes potential malignant transformation [65]. Senescence-associated secretory phenotype (SASP) turns senescent fibroblasts into proinflammatory cells that have the ability to promote tumor progression and inflammation-related pathologies. SASP cells secrete interleukins (IL-1 and IL-6), inflammatory cytokines (CXCL-8 and CCL), and growth factors (IGF) that can affect surrounding cells. In frail individuals, miR-146a expression was lower compared to robust aged individuals [22], potentially leading to dysregulated inflammation and a proinflammatory state. This suggests that while miR-146a may play a protective role against excessive inflammation during aging, its downregulation in frailty may contribute to an imbalance in inflammatory signaling pathways.

High concentrations of miR-223 and miR-483 were observed both in frail and robust populations [22], which indicates their relationship with aging in general but makes it difficult to qualify them as frailty biomarkers.

In an attempt to understand the linkage between frailty and the 8 selected miRNAs in [43]—miR-10a-3p, miR-92a-3p, miR-185–3p, miR-194–5p, miR-326, miR-532–5p, miR-576–5p, and miR-760— the authors performed pathway analysis using the Kyoto Encyclopedia of Genes and Genomes (KEGG) database. The top results showed mostly cancer, aging, and inflammation-related pathways (insulin resistance and signaling pathways; MAPK or Ras signaling pathways). The 3 most common matches of all 8 miRNAs were pathways related to cancer, the Ras signaling pathway, and insulin resistance. Although the study provides a very interesting insight into potential miRNA biomarkers, a significant limitation appears to be the very small study population.

MiR-151a-5p, miR-181a-5p, and miR-1248 play important roles in the aging process by influencing inflammatory regulation, DNA repair, and metabolic pathways [44]. However, their direct involvement in frailty syndrome remains insufficiently explored, and the population size is small. While existing evidence suggests a potential link between these miRNAs and frailty-related physiological decline, further research is needed to establish their precise role as biomarkers or mediators of frailty progression.

#### 3.1.2. Long Noncoding RNA

Several studies have identified lncRNAs involved in muscle regeneration and age-related dysfunction, suggesting their relevance in assessing frailty. For specific lncRNAs, *linc-MD1* plays a crucial role in early myogenesis by interacting with RNA-binding proteins, affecting muscle differentiation and regeneration [67]. Similarly, *Malat1* has been shown to be inhibited by myostatin, leading to decreased myogenesis, which may contribute to muscle loss and frailty in aging populations [68]. Additionally, myostatin-mediated dysregulation of muscle stem cells has been linked to *lncRNA* involvement in sarcopenia, a key feature of frailty [69]. Genetic variations in the *p16(INK4a)* locus have been associated with physical function decline in older individuals, indicating that regulatory elements, including lncRNAs, may play a role in frailty progression [70]. Additionally, genome-wide association studies have linked the 9p21-23 locus to frailty, suggesting a broader genetic basis for this syndrome beyond sarcopenia alone [46]. The ANRIL lncRNA, located at the 9p21 genetic locus, has been identified as a key hotspot in genome-wide association studies related to cardiovascular disease risk [71]. While ANRIL dysregulation at the 9p21 locus is an interesting finding, its clinical utility as a frailty biomarker remains uncertain due to the limited sample size, lack of longitudinal validation, and unclear causality (it remains uncertain whether ANRIL expression changes drive frailty or if frailty itself leads to altered ANRIL levels). Given the known interplay between frailty and cardiovascular diseases, further studies are needed to explore whether lncRNAs like *ANRIL* could serve as dual-purpose biomarkers for both conditions.

#### 3.1.3. DNA Methylation

Global DNA hypomethylation has been found to correlate with frailty, suggesting a potential role in age-related functional decline. On the other hand, telomere length measured in leukocytes, a well-studied parameter related to aging, was not associated with frailty [61,62].

Interestingly, no significant differences in methylation levels were observed among ultranonagenarians (individuals aged 90 and above), regardless of their frailty status, indicating that the association between DNA methylation and frailty may diminish in extreme old age [51]. Additionally, neither the frailty index nor any DNAm age estimators were significantly associated with chronological age in the Italian population of semi-supercentenarians [60], possibly due to the extreme heterogeneity in health status within this age group. The Yakumo Study [52] examined LINE-1 methylation levels in older adults and found that men over 60 years old with sarcopenia had significantly lower LINE-1 methylation levels compared to non-sarcopenic individuals. However, no significant differences were observed in women, suggesting a possible sex-specific association [72]. In contrast, the Newcastle 85+ Study investigated the relationship between DNA methylation and frailty in very old individuals and found that while lower promoter-specific CpG island methylation was associated with increased frailty, genome-wide methylation levels measured via LINE-1 showed no significant correlation with frailty. This suggests that specific gene regulatory regions may be more relevant as biomarkers for frailty than global methylation levels [53].

#### 3.1.4. EWAS

Hypomethylation at CpG sites identified by Kim et al. (2018) [47] was associated with increased frailty scores, indicating their potential role as epigenetic markers of frailty. These sites—cg19283806 (KCNQ1), cg21572722 (PHF21A), and cg05575921 (AHRR)—are linked to pathways involved in inflammation, oxidative stress, and metabolic regulation, all of which are known contributors to frailty. The study suggests that these loci could serve as valuable markers for early detection of frailty risk. EWAS performed by Li et al. (2022) [49] identified 65 frailty-associated CpGs, with 20 of them selected to construct the epigenetic Frailty Risk Score (eFRS). The eFRS demonstrated strong predictive power for both prevalent and incident frailty over long-term follow-up, with validation in independent cohorts. Additionally, the correlation between eFRS and GrimAge observed in the study highlights a shared epigenetic signature between frailty and biological aging, suggesting that DNAm-based markers could enhance early frailty risk assessment and intervention strategies. Among the identified CpG sites, 12 were annotated to genes involved in chronic conditions such as coronary artery disease, stroke, and type 2 diabetes mellitus, reinforcing the connection between frailty and broader age-related diseases. Gale et al. (2018) [48] examined the Lothian Birth Cohort 1936—their findings identified a CpG site located in the MAF1 gene (cg18314882) that was associated with frailty status. MAF1 is a key regulator of RNA polymerase III and is involved in metabolic regulation and cellular stress responses, pathways known to influence aging and frailty. Notably, the CpG site identified in this study did not overlap with those found in the EWAS by Li et al. (2022) [49] or Kim et al. (2018) [47], suggesting that multiple distinct epigenetic mechanisms may contribute to frailty. The most recent study by Mak et al. (2024) [50] integrated findings from four previous EWAS analyses, further confirming the role of DNA methylation in frailty and aging. This large-scale analysis identified several CpG sites associated with both frailty and cardiovascular disease, reinforcing the interconnection between aging-related epigenetic changes and systemic health decline. Notably, the study highlighted GrimAge and PhenoAge as robust predictors of frailty and health outcomes, demonstrating their ability to capture cumulative epigenetic modifications linked to functional decline. These findings underscore the potential of DNAm-based biomarkers not only for frailty risk assessment but also for broader age-related disease prediction.

#### 3.1.5. Epigenetic Clocks

Among epigenetic clocks, the strongest correlations with frailty were observed for those designed to capture phenotypic health markers and aging-related mortality, particularly GrimAge and PhenoAge. These clocks were not only linked to baseline frailty status but also to longitudinal changes over time [54,55]. However, their predictive value varied across different frailty phenotypes and all-cause mortality. However, their predictive value varied across clinical frailty phenotypes and all-cause mortality. In the study by McCrory et al. [39]. HorvathAA and HannumAA were not reliable predictors of health outcomes, while PhenoAgeAA was initially associated with four out of nine frailty-related measures (walking speed, frailty, Montreal Cognitive Assessment, and Mini-Mental State Examination) but lost significance after adjustment for social and lifestyle factors. In contrast, GrimAgeAA showed associations with eight out of nine outcomes (all except grip strength) in minimally adjusted models and remained significantly correlated with walking speed, polypharmacy, frailty, and mortality even after full adjustment. Wang et al. [56] further confirmed the predictive value of PhenoAge and GrimAge for mortality, with MRscore-8CpGs emerging as the strongest predictor among all assessed clocks. Similarly, findings from the China Kadoorie Biobank study [57] demonstrated that GrimAgeAA was significantly associated with frailty and mediated the effects of various metabolic and inflammatory biomarkers. While first-generation clocks (HorvathAA, HannumAA, LiAA) were primarily linked to cardiovascular aging and atherosclerotic cardiovascular disease risk, GrimAgeAA stood out as the most robust predictor of frailty, further reinforcing its potential as a key biomarker for aging-related functional decline.

Despite such promising perspectives, many studies do not exhibit a similar relationship between frailty and epigenetic clocks. The MOBILIZE Boston study [59] results indicate that there was no consistent association between eAA and changes in frailty over time. While baseline frailty showed some associations with PhenoAge and GrimAge eAA, these relationships did not persist when assessing frailty progression. Consistently, Kim et al. [40] demonstrated that the frailty index outperforms DNA methylation age and its derivatives as an indicator of biological age, reinforcing the importance of clinical frailty assessments over epigenetic clocks in predicting health outcomes. Similarly, the BASE-II study [61] found no consistent relationship between various epigenetic clocks and functional assessments in older adults. Although PhenoAge and GrimAge acceleration were weakly associated with some physical and cognitive measures, these associations did not remain significant when adjusting for covariates. Interestingly, GrimAge acceleration required an increase in more than 50 years to correspond to a one-point increase in Fried’s frailty score, suggesting that its effect size in relation to frailty is relatively small. The Italian semi-supercentenarian study [60] further challenges the utility of epigenetic clocks in frailty assessment by demonstrating no association between DNA methylation age estimators and frailty in individuals aged 104–109 years. This may be due to the highly heterogeneous health status of centenarians, where extreme longevity and unique resilience mechanisms obscure typical aging patterns. In very old individuals, frailty may be better reflected by multidimensional clinical biomarkers rather than molecular aging markers, as the latter may no longer adequately differentiate between biological aging trajectories at such advanced ages.

In contrast to these findings, the study on temporal dynamics of epigenetic aging and frailty [58] suggests that while first- and second-generation clocks do not exhibit dynamic coupling with frailty over time, DunedinPACE—an epigenetic measure of the pace of aging—does. The unidirectional relationship observed indicates that increases in DunedinPACE precede frailty progression, supporting the geroscience hypothesis that interventions targeting biological aging could delay frailty onset. DunedinPACE’s ability to capture ongoing physiological deterioration may explain its stronger predictive value compared to traditional clocks, which primarily estimate cumulative biological age rather than real-time aging processes.

The study by Gao et al. (2017) [63] found that current smoking, cumulative smoking exposure (measured in pack-years), and time since smoking cessation were all significantly correlated with FI. Current smokers had a higher frailty burden compared to former smokers and non-smokers, suggesting that smoking accelerates health deficits contributing to frailty. Interestingly, the smoking index (SI), derived from methylation patterns, exhibited a stronger association with frailty than self-reported smoking status, emphasizing the potential of DNA methylation markers in objectively assessing smoking-related health risks. Among the identified CpG sites, three were linked to genes involved in chronic inflammation (MYO1G, GPR15, and GNG12), a known driver of frailty progression. Three other CpGs were associated with genes implicated in age-related cancers, while one CpG site was previously associated with obesity, a key frailty component. The study’s findings are particularly relevant for CVD risk evaluation, as smoking is a well-established risk factor for cardiovascular diseases, and its effects on DNA methylation could serve as an important biomarker for both frailty and CVD, and the DNA methylation-based measures of smoking exposure may be useful for predicting long-term health outcomes and guiding targeted interventions to reduce frailty and CVD risk.

### 3.2. Screening for Cardiovascular Relevance

Ekerstad et al. [73] found that age was the only significant difference between frail patients with myocardial infarction who did or did not undergo coronary angiography (mean age 80 vs. 86), though the age ranges overlapped, preventing a clear cut-off. This study was among the first to show that only a minority of frail patients received angiography, and fewer than half underwent revascularization. More recent data show an upward trend, with 56.5% of frail patients receiving PCI for acute coronary syndrome between 2004 and 2021 period [74]. All without-exclusion papers document that frail patients treated with PCI, irrespective of primary diagnosis, e.g., acute or stable coronary syndrome, have an increased risk of periprocedural bleeding [10,75], vascular injury [75], stroke or transient ischemic attack [75], and short-, medium-, and long-term mortality risk [13,73,74,75,76]. He et al. [3] reported significantly increased odds of death for frail patients undergoing PCI: 3.59 in-hospital, 6.61 medium-term, and 3.24 long-term. Coronary bypass surgery shows similar risks, with nearly half of frail patients experiencing increased mortality or institutional discharge [77,78]. Freiheit et al. [79] offered further insight by analyzing outcomes in frail CAD patients across different treatment arms (medical therapy, PCI, CABG). Their findings suggest that younger patients (<75 years) undergoing PCI or CABG experienced temporary improvements in frailty status, unlike their older counterparts. Chen et al. (2024) [80] highlighted strong links between genetic and phenotypic frailty, cardiovascular traits, and behavior, suggesting that genetic risk integration may enhance early identification of vulnerable patients. These findings emphasize the importance of reproducible biomarkers in frailty assessment. Among many candidates, the most relevant biomarkers were selected for analysis. While some primarily reflect aging and require further validation, most existing research focuses on microRNAs and DNA methylation, with no direct studies on histone modifications or other epigenetic changes in frailty.

Frailty and cardiovascular diseases share common underlying mechanisms, including chronic inflammation, oxidative stress, and metabolic dysregulation. As a result, biomarkers of frailty not only reflect age-related functional decline but also provide valuable insights into cardiovascular health and disease risk. Several molecular biomarkers have emerged as potential indicators of both frailty and CVD, offering a deeper understanding of their shared pathophysiology.

Among microRNAs, miR-21, miR-146a, miR-451, and miR-92a have been implicated in processes such as endothelial dysfunction, inflammation, and vascular remodeling, linking them to various cardiovascular conditions. Global DNA methylation patterns, as reflected by LINE-1 methylation levels, have also been explored in relation to both frailty and CVD, particularly in the context of atherosclerosis and vascular aging. Additionally, epigenetic clocks such as GrimAge and general epigenetic age acceleration (eAA) have demonstrated predictive value for cardiovascular risk, reinforcing the role of epigenetic mechanisms in aging-related diseases. Specific frailty-related epigenetic markers, including the epigenetic frailty risk score (eFRS) and the Smoking Index, further highlight the interplay between frailty, metabolic dysfunction, and cardiovascular pathology, with strong associations observed for conditions such as coronary artery disease, heart failure, and stroke.

A summary of the key findings on the relationship between the most promising frailty biomarkers and CVD is presented in Table 2.

**Table 2:** Cardiovascular relevance of selected frailty biomarkers.

Epigenetic Factor	Key Findings	Reference Articles
**MiR-21**	Mediates macrophage-to-fibroblast signaling in heart failure cardiac remodeling. Up-regulated in human atherosclerotic plaques. Elevated in acute myocardial infarction. AntimiR-21 treatment prevented myocardial dysfunction and reduced pathologic remodeling of the heart in animal models. Increased significantly in response to acute exhaustive exercise in patients with congestive heart failure.	[26,81,82,83,84,85,86,87,88,89]
**MiR-451a**	Elevated in acute myocardial infarction. Regulates oxidative stress and redox homeostasis. Associated with atrial fibrillation progression; potential biomarker for recurrence risk after cardioversion.	[90,91,92]
**Mir-146**	Up-regulated in human atherosclerotic plaques. Increased coronary heart disease in patients with subclinical hypothyroidism. Modulates endothelial function and monocyte-macrophage activity; negatively correlated with TNF-α levels in stable coronary artery disease. Plays a key role in cardiac regeneration and repair by modulating inflammatory responses and fibrosis in cardiovascular disease. Modulates pro-inflammatory and fibrotic signaling pathways in diabetic cardiomyopathy.	[83,91,92,93,94,95,96]
**Mir-92a**	Regulates myocardial redox balance by reducing oxidative stress, providing cardioprotective function. Inhibits endothelial cell angiogenesis both in vitro and in vivo. Identified as a mechano-sensitive microRNA highly expressed in endothelial cells, its expression increases under pro-inflammatory shear stress and exposure to oxidized LDL.	[26,97,98]
**LINE-1** **methylation**	Lower methylation levels are associated with increased risk of coronary heart disease and myocardial infarction. Lower methylation levels are associated with higher LDL and lower HDL levels.	[99,100,101]
**GrimAge (eAA)**	GrimAge acceleration is significantly associated with cardiometabolic risk factors and elevated clinical cardiovascular disease risk scores. Epigenetic age acceleration based on GrimAge is linked to an increased risk of incident atrial fibrillation, general increased CVD risk, arterial stiffness, and adverse cardiac remodeling. GrimAge acceleration correlates with early-life plasma lipid profiles and is associated with both single-site and multi-site atherosclerosis. GrimAge and PhenoAge predict the development of atherosclerotic cardiovascular disease through their mediation of metabolic alterations and epigenetic aging. Epigenetic age acceleration is correlated with general increased CVD risk, arterial stiffness and adverse cardiac remodeling.	[50,57,102,103,104,105,106,107,108]
**eFRS**	Few assessed CpGs overlap with known cardiovascular disease markers; shared pathways between frailty and cardiovascular risk.	[49]
**Smoking Index**	Smoking is a key risk factor for CVD, and its associated methylation changes overlap with those linked to inflammation and aging.	[63]

Despite inconsistencies and variability in the analyzed literature, GrimAge has shown the most promise in its correlation with frailty syndrome and surrogate aging factors. Calculation of GrimAge involves DNAm surrogates for adrenomedullin, β-2-microglobulin, cystatin C, growth differentiation factor-15, leptin, PAI-1, tissue inhibitor metalloproteinases-1, and smoking pack-years, according to the original studies. Many of those proteins are connected to age-related pathologies, including inflammation, hypertension, heart failure, kidney function, and cognitive functioning [42]. It is not a surprise that GrimAges scores correlate strongly with cardiovascular risk [102,105,109] and many different cardiovascular diseases, including atrial fibrillation [106], atherosclerosis, and dyslipidemia [103,104]. The study by Si et al. [57] demonstrated that GrimAge and PhenoAge DNA methylation clocks mediate the relationship between circulating metabolites and the development of atherosclerotic cardiovascular disease and frailty, reinforcing the role of epigenetic aging markers in assessing cardiovascular risk in frail individuals. It was the only study that was directly linking CVD and frailty. While specific CpG sites and genes identified in different EWAS cohorts do not fully overlap, their involvement in metabolic, inflammatory, and oxidative stress pathways underscores the complex biological underpinnings of frailty. The integration of DNAm-based scores such as eFRS with established aging clocks (GrimAge, PhenoAge) may improve the accuracy of frailty assessment and facilitate earlier intervention strategies.

The lack of association between epigenetic clocks and frailty in some studies [39,59,60,61] may be attributed to several factors: First, the study population consisted of relatively healthy older adults [61], with a lower-than-expected prevalence of frailty compared to similar cohorts, which may have limited the ability to detect meaningful associations. Second, epigenetic clocks primarily reflect molecular aging rather than accumulated health deficits, which are central to frailty assessment. As a result, they may not effectively capture functional decline. Third, differences between epigenetic clocks could explain the variability in findings. While first-generation clocks (Horvath, Hannum) were designed to estimate chronological age, second-generation clocks (GrimAge, PhenoAge) incorporate clinical risk factors such as inflammation and smoking, which may explain GrimAge’s better association with frailty. Finally, frailty is a complex syndrome influenced by multiple biological pathways, and epigenetic aging alone may not fully explain its development. The heterogeneity in aging trajectories suggests that additional biomarkers, beyond DNA methylation-based aging clocks, may be necessary for accurately predicting frailty progression. Another hypothesis suggests reverse causation, wherein frailty itself—through factors such as inflammation and metabolic dysregulation—may drive epigenetic changes, rather than being a direct consequence of epigenetic aging. Future research should focus on refining epigenetic biomarkers that more directly reflect functional decline, incorporating longitudinal designs with extended follow-up periods, and exploring novel metrics such as DunedinPACE that may better capture the dynamics of frailty progression [58].

A valuable direction for future research is the exploration of CVD biomarkers that share similar biological mechanisms with those used in frailty assessment. For instance—DNAmCVD score [102], a blood-based DNA methylation biomarker designed to predict short-term cardiovascular events, demonstrated superior predictive performance compared to existing models, including GrimAge, particularly for events occurring within a seven-year follow-up period. Currently, there are no direct studies investigating the application of the DNAmCVD score as a biomarker in frailty populations. However, the DNAmCVD score has been developed as a DNA methylation-based biomarker, similar to many others discussed above, which suggests potential utility in older and frail populations, where cardiovascular risk is elevated. Further research is needed to validate its applicability in these specific groups.

### 3.3. Final Considerations

We have challenged the task of integrating two complex clinical conditions—frailty and cardiovascular diseases—to identify biomarkers that could serve as potential candidates for clinical application, particularly in cardiology. The goal was to find the epigenetic biomarkers for enhanced risk assessment and tailored treatment strategies and evaluate potential benefits for patients at risk of both conditions. Compared to conventional clinical scores, epigenetic biomarkers offer a more individualized and biologically grounded perspective, capturing early molecular changes associated with aging and disease progression; this may improve risk stratification, particularly in borderline or heterogeneous cases where traditional tools lack sensitivity. However, the cost and technical requirements of these methods may be challenging for routine clinical use, especially in settings with limited resources, though they do not rule out future use as technology improves and becomes more affordable.

In clinical practice, both microRNA and DNA methylation biomarkers have shown significant promise in detecting and monitoring frailty and cardiovascular risk, yet each approach carries distinct advantages and limitations. On one hand, miRNAs are readily accessible in blood and can reflect dynamic changes in inflammation, metabolism, and other CVD- and frailty-related processes [21,22,23,110]. They are generally easier to sample repeatedly, and emerging standardized protocols are gradually improving assay reliability [87]. Current evidence suggests that miRNAs could serve not only as diagnostic tools but also as dynamic biomarkers to monitor therapeutic response or disease progression over time. However, multiple miRNAs often overlap in function, complicating the interpretation of specific signaling pathways. Additionally, factors such as diet, time of day, stress, medications, and comorbidities may alter miRNA levels, potentially reducing their specificity in large, heterogeneous patient populations [111,112,113]. For instance, despite being the most profoundly analyzed miRNA in frailty assessment, miR-21 has been identified as a highly nonspecific biomarker, with elevated expression observed across various pathological conditions, including cardiovascular diseases, cancer, kidney disease, and autoimmune disorders [112]. This broad association significantly limits its utility as an independent diagnostic marker for any single condition. Instead, its upregulation often reflects a general pathological state rather than a disease-specific signature.

On the other hand, DNAm-based measures, including global LINE-1 methylation and epigenetic clocks, provide a more stable snapshot of biological aging and are strongly predictive of frailty outcomes [39,54,55]. Yet, these methods typically require specialized equipment and advanced bioinformatic tools, limiting accessibility in routine clinical settings. Tissue specificity remains another concern: while peripheral blood DNAm correlates with systemic aging processes, it may not fully capture organ-specific frailty mechanisms. Many studies [40,58,59,60,61] noted that DNAm-based clocks, while effective in predicting biological age, often fail to capture the multidimensional aspects of frailty compared to traditional measures such as the frailty index. Moreover, longitudinal assessment of DNAm patterns can be costly, and high inter-laboratory variability persists in measuring site-specific methylation. This raises important questions about the feasibility of implementing DNAm clocks like GrimAge in everyday cardiology practice. Key requirements for their clinical adoption include cost reduction, automation of laboratory protocols, improving access to sequencing technology, and creating standardized methods for data analysis. Barriers to implementation include high initial investment in equipment, the need for trained personnel, and the current lack of clinical guidelines integrating epigenetic data into cardiovascular risk stratification.

Despite these differences, both miRNA- and DNAm-based biomarkers share a key benefit: the ability to detect preclinical changes in frailty trajectories. By refining current protocols and addressing assay standardization, these approaches may become more robust. Ultimately, combining conventional frailty assessments, miRNA, and DNAm methods could provide a more comprehensive view of aging biology, facilitating optimal risk stratification and therapeutic decision-making in older adults with cardiovascular disease. Therefore, we advocate for the development of a multi-biomarker panel that could improve diagnostic precision. Such panels would need to be benchmarked against established clinical tools like the frailty index or the SCORE system to determine their relative or added predictive value.

Recent works suggest that epigenetic aging is potentially reversible, so the epigenetic clocks may also represent a means for quantifying the efficacy of interventions designed to retard or reverse the aging process [114]. Targeted interventions, such as lifestyle modifications and pharmacological strategies, have the potential to mitigate frailty progression by addressing its underlying biological mechanisms, including chronic inflammation and metabolic dysregulation [111]. Physical activity is recognized as an effective intervention for frailty, improving muscle strength, cardiovascular function, and reducing systemic inflammation, thereby mitigating age-related decline [115]. In diseases, particularly ischemic heart disease, structured exercise programs may offer a safer and more sustainable therapeutic approach compared to pharmacological treatments, enhancing myocardial perfusion and reducing cardiovascular risk without the side effects associated with conventional therapies.

In the scoping review process, despite careful selection and evaluation of studies, several limitations may arise. First, due to incomplete data from some studies, there is a possibility that certain results were omitted or unpublished, which could affect the interpretation of the overall findings. Another limitation is that some analyses were based on studies with varying methodological quality, which might introduce bias in assessing the effectiveness of the analyzed biomarkers. Additionally, due to the nature of the review, which relies on available primary studies, the ability to account for certain important factors, such as environmental variables or differences in study protocols, was limited. Furthermore, no statistical or computational analyses were employed in this review, limiting the ability to conduct more advanced synthesis or examine potential interactions between variables in a quantitative manner.

## 4. Conclusions

At present, there are no published studies directly investigating the use of epigenetic biomarkers for cardiovascular risk stratification in frail patients. Our review indicates that potential biomarkers should primarily be sought among microRNAs and DNA methylation markers. Among miRNAs, the most promising candidates are miR-146a, miR-451, miR-92a, and miR-21; however, due to their limited specificity, especially in the case of miR-21, these should not be considered standalone biomarkers. Regarding DNA methylation-based approaches, LINE-1 and GrimAge appear to be the most relevant, although existing evidence presents some inconsistencies. Future research should explore emerging epigenetic clocks, such as DunedinPACE and DNAmCVDscore, which may provide enhanced predictive accuracy for both frailty and cardiovascular risk. Additionally, the Smoking Index merits further evaluation as a potential biomarker, given its strong association with both frailty and cardiovascular disease risk.

## Data Availability

Data and materials used in this review, including data extracted from the included studies, are not publicly available, and no analytic code was utilized or provided for the analysis.

## Supplementary Materials

The following supporting information can be downloaded at: https://www.mdpi.com/article/10.3390/cimb47060422/s1. PRISMA_2020_abstract_checklist and PRISMA_2020_checklist [116].

## Abbreviations

The following abbreviations are used in this manuscript:

CVD	cardiovascular disease
PCI	percutaneous coronary intervention
CABG	coronary artery bypass graft
FI	frailty index
GRACE	Global Registry for Acute Coronary Events
REPOSI	REgistro POliterapie SIMI (Registro Politerapie Società Italiana di Medicina Interna)
PRISMA	Preferred Reporting Items for Systematic Reviews and Meta-Analyses
EWAS	epigenome-wide association study
CpG	Cytosine-phosphate-Guanine
eFRS	epigenetic Frailty Risk Score
DMRs	differentially methylated regions
LINE-1	Long Interspersed Nuclear Element-1
DNAm	DNA methylation
eAA	epigenetic Age Acceleration
SASP	senescence-associated secretory phenotype
miRNA	microRNA
lncRNA	long noncoding RNA
ANRIL	antisense noncoding RNA in the INK4 locus
TLRs	toll-like receptors
NF-κB	nuclear factor kappa-light-chain-enhancer of activated B cells
IL	interleukin
TNF-α	tumor necrosis factor-alpha
TGF-β	transforming growth factor-beta
IRAK1	interleukin-1 receptor-associated kinase 1
TRAF6	TNF receptor-associated factor 6
KEGG	Kyoto Encyclopedia of Genes and Genomes
Ago2	Argonaute 2
LDL	low-density lipoprotein
HDL	high-density lipoprotein
CRP	C-reactive protein
PAI-1	plasminogen activator inhibitor-1
GDF15	growth differentiation factor 15
SI	Smoking Index
DNAmCVDscore	DNA methylation cardiovascular disease score
MAPK	mitogen-activated protein kinase
Ras	Rat sarcoma (small GTPase proteins)
mRNA	messenger RNA
AUC	area under the curve
HR	hazard ratio
OR	odds ratio
